# Measuring Coverage in MNCH: Determining and Interpreting Inequalities in Coverage of Maternal, Newborn, and Child Health Interventions

**DOI:** 10.1371/journal.pmed.1001390

**Published:** 2013-05-07

**Authors:** Aluísio J. D. Barros, Cesar G. Victora

**Affiliations:** Postgraduate Program in Epidemiology, Federal University of Pelotas, Pelotas, Brazil; Professor of Demography and Social Statistics, University of Southampton, United Kingdom

## Abstract

To monitor progress towards the Millennium Development Goals, it is essential to monitor the coverage of health interventions in subgroups of the population, because national averages can hide important inequalities. In this review, we provide a practical guide to measuring and interpreting inequalities based on surveys carried out in low- and middle-income countries, with a focus on the health of mothers and children. Relevant stratification variables include urban/rural residence, geographic region, and educational level, but breakdowns by wealth status are increasingly popular. For the latter, a classification based on an asset index is the most appropriate for national surveys. The measurement of intervention coverage can be made by single indicators, but the use of combined measures has important advantages, and we advocate two summary measures (the composite coverage index and the co-coverage indicator) for the study of time trends and for cross-country comparisons. We highlight the need for inequality measures that take the whole socioeconomic distribution into account, such as the relative concentration index and the slope index of inequality, although simpler measures such as the ratio and difference between the richest and poorest groups may also be presented for non-technical audiences. Finally, we present a framework for the analysis of time trends in inequalities, arguing that it is essential to study both absolute and relative indicators, and we provide guidance to the joint interpretation of these results.

## Abstract 2

In a *PLOS Medicine* Review, Aluísio Barros and Cesar Victora provide a practical guide to measuring and interpreting inequalities in the coverage of maternal, newborn, and child interventions in low- and middle-income countries using data collected by large household surveys.

*This paper is part of the* PLOS Medicine *“Measuring Coverage in MNCH" Collection*

## Introduction

Equity in health has been part of the public health agenda for quite some time in the US, Europe, and Latin America [1]–[3], but interest in health inequities has boomed since the 1990s, with a large number of publications considering definitions [3], measurement [4]–[6], and controversies about health inequalities [7],[8] (throughout this review we will refer to equity when we are considering the concept of fairness/justice and inequality when we are considering the measurement of differences in coverage, which are used to make judgments about equity/inequity). Interest in health equity has also started to increase in low- and middle-income countries, where few analyses of inequalities were available prior to 2000 [9]–[11]. Although reducing inequalities was not a key element in the health-related Millennium Development Goals, it is an important focus of the post-2015 agenda, which involves studying how inequalities change, how they relate to policies and health systems, and how they relate to global processes, such as conflict or economic growth or recession [12]. The need to make a clear link between broad social and economic inequalities and disparities in the coverage of health interventions has also been championed by the Social Determinants of Health movement [13],[14].

In spite of recent developments, descriptive cross-sectional studies of health inequalities are still the most common and useful type of study for the design and implementation of public health policies aimed at improving equity. Such studies require the measurement, presentation, comparison, and interpretation of inequalities in health. In this article, which is part of the *PLOS Medicine* “Measuring Coverage in MNCH" Collection, we do not intend to provide a broad review of inequalities in health and their measurement, but rather we rely on our own recent experience in monitoring inequalities [15] to provide practical advice to researchers and policymakers in low- and middle-income countries on how to carry out and interpret such analyses. We discuss methodological issues relevant to these objectives, including the assessment of socioeconomic position, choice of outcome measures, measures of the degree of inequality, and assessment of changes in inequalities over time. The examples we include are derived from data analyses carried out for the World Health Organization Global Health Observatory [16] and for the Countdown to 2015: Maternal, Newborn and Child Survival initiative [17]. Our examples use primary data mainly collected by the Demographic and Health Surveys (DHS) [18] and Multiple Indicator Cluster Surveys (MICS) [19], large, population-based household surveys that have been carried out repeatedly in low- and middle-income countries since the 1990s. Comprehensive overviews of health inequalities based on data from these surveys are available elsewhere [20]–[22].

## Measuring Socioeconomic Position

There are multiple dimensions to health equity according to gender, wealth, education, place of residence, ethnicity, and sexual orientation, among other factors. In this article we focus on “socioeconomic position," a term that is now preferred over “socioeconomic status" in the equity literature [23].

Socioeconomic position can be ascertained using different types of indicators, each reflecting slightly—or sometimes markedly—different underlying constructs. From the standpoint of statistical analyses, an indicator should be easy to measure in a valid way during surveys, should not change rapidly over time, should allow breakdown into several categories (preferably of similar size), and should be comparable over time and place. No single measure of socioeconomic position fulfills all these criteria in a satisfactory way. Howe et al. have recently reviewed the advantages and limitations of socioeconomic position indicators in low- and middle-income countries [24], and we provide a brief discussion of four ways to measure socioeconomic position—education, income, household consumption, and occupation—in Box 1.

**Box 1. Common Measures of Socioeconomic Position**

**Education of the Mother**

- Easy to measure but can also have a direct effect on health [12].
- Often results in unbalanced groups. In poor countries, a large proportion of women may have no education, whereas in wealthier countries most will have completed secondary school.
- Size of the categories will vary over time, as more women are educated, which affects the comparison of time trends.
- May be difficult to use in country comparisons because of different schooling structure, level names, and content.

**Income**

- Requires several questions to be asked about different sources of income.
- Misreporting is frequent, and monthly variability may be important in low-income societies where casual labor and agricultural production are common.
- More positively, income is a continuous variable that can be broken down in groups of uniform size, which allows comparisons over time.

**Consumption Expenditure**

- Reflects what people spend rather than what they earn.
- Difficult to measure, requiring respondents to keep diaries and to answer long questionnaires, and requiring multiple visits by interviewers.
- Affected by misreporting, seasonality, in-kind exchanges, and domestic production of goods [24],[44],[45].
- If properly measured, consumption expenditure is a useful indicator, but its practical limitations have so far restricted its use in health research in low- and middle-income countries.

**Occupation**

- Commonly used in high-income country studies, this measure of socioeconomic position is problematic in low- and middle-income countries, where changes in occupation and multiple jobs are common and unemployment or informal jobs predominate.
- Long questionnaires and complex post-processing are required to capture all the subtleties of occupation in low- and middle-income countries, where large proportions of the population may fall into a single category. “Farmers," for example, may include anyone from a landless laborer to a plantation owner.
- Several classifications used in countries are not ordinal, making it impossible to rank groups.

In light of the problems with the above socioeconomic position indicators, an alternative was proposed by Filmer and Pritchett in 1998 [25]—the asset index. This index is based on a relatively short list of household possessions (radio, television, refrigerator, etc.) and characteristics of the house (building materials, toilet, electricity, etc.), and may include the educational attainment of household members. These variables, which are collected in DHS and MICS surveys, are subjected to principal component analysis, a data reduction technique that produces a single continuous composite score from all the variables that retains as much variance as possible [26]. Each household is then assigned an asset score, and samples can be broken down into quintiles or other equal-sized groups of households based on this asset score.

Like all other socioeconomic position indicators, asset indices have limitations [27]. First, different choices of assets can change the classification of families [28],[29]. Second, families in the wealthiest quintile in most low- and middle-income countries are mainly urban [30], so that wealth inequalities are closely associated with urban/rural disparities [31]. A third limitation is that quintiles assess only relative socioeconomic position, rather than absolute socioeconomic position [32]. For example, the poorest quintile in a middle-income country may be richer than one of the wealthier quintiles in a low-income country. A similar problem can arise in time-trend analysis for a country that is getting richer. Moreover, because fertility tends to be higher among the poor, there tend to be more than 20% of the mothers and children in the lowest quintile of household wealth, and fewer than 20% in the richest quintile. This effect is even more marked for disease episodes: data on oral rehydration therapy, for example, are often based on a much larger sample (which is dependent on the number of diarrhea episodes) in the poorest than in the wealthiest quintile.

These limitations do not, however, preclude the widespread and valid use of asset indices for documenting the wide gaps between rich and poor that are present in most low- and middle-income countries, as is evident by the consistent associations between asset indices and more complex measures of socioeconomic position [31] and by the marked inverse associations between asset indices and child mortality and undernutrition [20],[21]. Compared to the other methods discussed above, the asset index has clear advantages, and this has resulted in its widespread international adoption as the preferred way of measuring socioeconomic position in low- and middle-income countries [20].

## Measuring Inequalities in Intervention Coverage

There are two basic approaches for measuring inequalities in intervention coverage. The first is to carry out separate analyses for each relevant coverage indicator, such as contraceptive use, presence of skilled birth attendant, measles vaccine coverage, oral rehydration therapy, etc. The Countdown to 2015 initiative [17] provides inequality breakdowns of 18 such indicators for 75 countries. There are at least two caveats with this approach. First, several coverage indicators are based on small subsets of mothers or children. For example, vaccination status is assessed among children aged 12–23 months, and oral rehydration therapy is assessed among children who presented with diarrhea in the two weeks before the interview. When these indicators are broken down by quintiles, the number of mothers and children surveyed who (a) belong to a given quintile and (b) also belong to the subgroup that constitutes the denominator of the coverage indicator is often very small, even in large surveys, which leads to poor precision due to sampling variability. The second caveat is the difficulty in summarizing inequalities for a given country, because the magnitude of inequality may vary by indicator. On the other hand, these analyses may provide insights about which delivery channels are most equitable, and therefore contribute to better programming. For example, a recent Countdown to 2015 publication compared inequalities in coverage with skilled birth attendants—which requires access to a functioning health system, 24 hours a day and seven days a week—and inequalities in measles vaccine coverage—which is usually delivered on a single occasion in communities, often during national immunization days. Not surprisingly, inequalities were much greater for coverage with skilled birth attendants than for coverage with measles vaccine [22].

To avoid the problems of studying one coverage indicator at a time, two related measures have been proposed that combine the coverage of several interventions (Box 2). The co-coverage indicator is important since it shows what percentage of the population is receiving all, or most, of the main preventive interventions. If children or mothers receive just a few of a set of lifesaving interventions, efforts to improve the health of children or mothers may have little effect [33]. The composite coverage index (CCI), on the other hand, provides an overall estimate of coverage based on eight essential health interventions. By replacing a large number of coverage estimates, it makes multi-country and time-trend assessments easier to carry out and to understand. Both measures have been widely used in cross-country comparisons and in equity analyses; the most recent Countdown to 2015 report contains several examples [34].

**Box 2. Indicators That Combine Coverage of Several Interventions**

**Co-Coverage**

**Figure 1:**
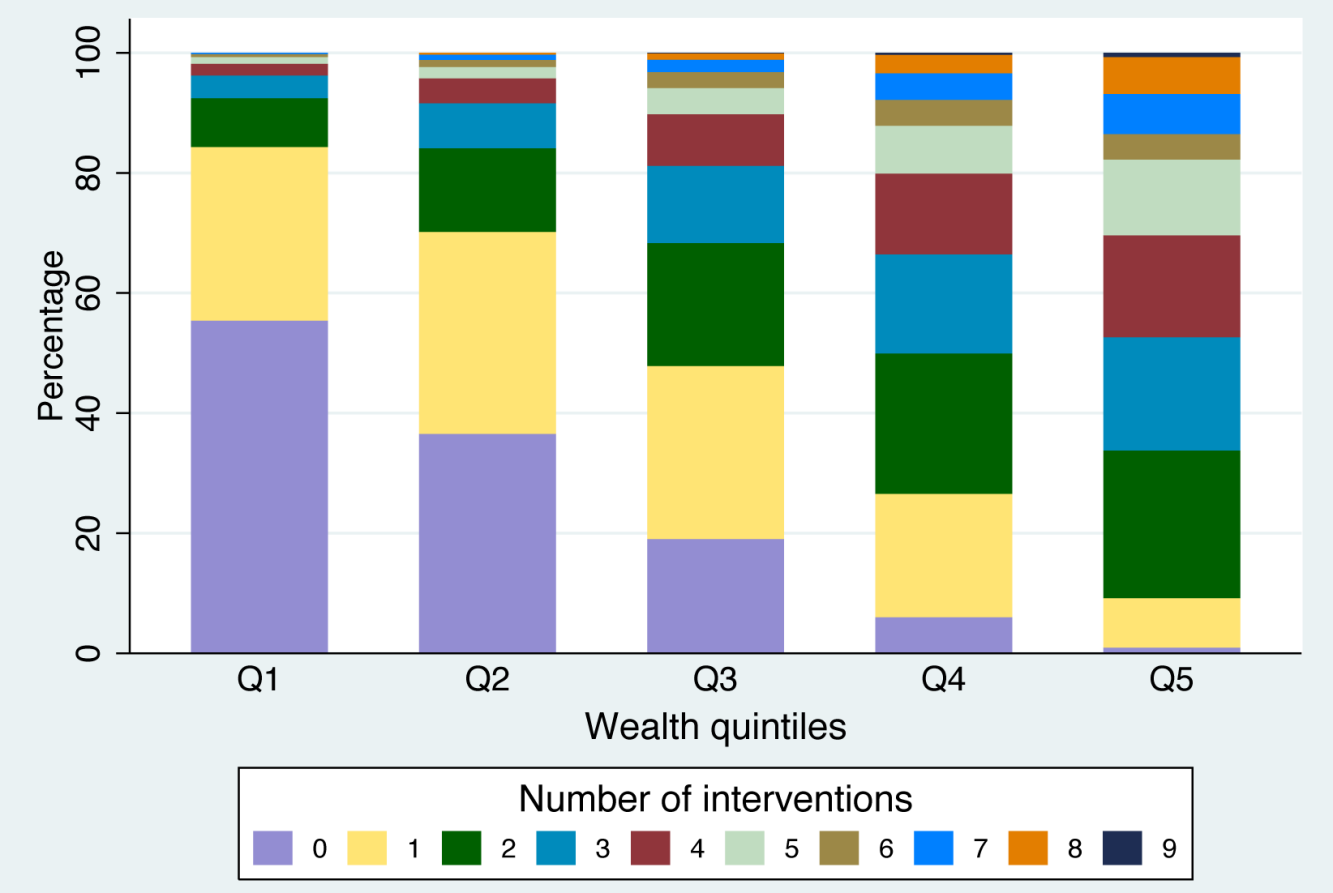
Co-coverage of nine preventive interventions for Nigeria (DHS 2008), by wealth quintiles. See Box 2 for more information on the interventions included.

- Based on how many preventative interventions each mother/child pair received, out of a set of 8–9 essential interventions [33]: antenatal care (1+ visit with skilled provider); tetanus toxoid during pregnancy; skilled birth attendant; child received vitamin A supplementation, BCG (tuberculosis) vaccination, DTP3 (diphtheria–tetanus–pertussis) vaccination, and measles vaccination; improved drinking water source. Insecticide-treated bednets are also included in countries where relevant.
- Calculation of co-coverage requires reanalysis of original survey data, which is time-consuming, but because co-coverage is measured at the individual level, standard errors and confidence intervals can be calculated.
- Co-coverage is often reported as the percentage of children covered by at least three or six interventions but can also be presented through stacked bar graphs that show the percentage of children in the population covered by a given number of interventions, usually stratified by wealth quintiles (Figure 1).

**Composite Coverage Index (CCI)**

- Based on the weighted average of coverage of a set of eight preventative and curative interventions; the CCI gives equal weight to four stages in the continuum of care: family planning, maternal and newborn care, immunization, and case management of sick children [27].
- The weighted average for a group (e.g., a country or a wealth quintile) is calculated as
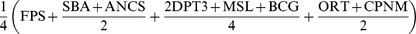
where FPS is family planning needs satisfied, SBA is skilled birth attendant, ANCS is antenatal care with skilled provider, DPT3 is three doses of diphtheria–pertussis–tetanus vaccine, MSL is measles vaccination, BCG is BCG (tuberculosis) vaccination, ORT is oral rehydration therapy for children with diarrhea, and CPNM is care seeking for pneumonia.
- Because the CCI is a group indicator, jackknife or similar resampling methods are required to estimate its standard error [46].

## How to Express the Magnitude of Inequalities

There is no consensus on the ideal measure for expressing the magnitude of inequalities. In 1991, Wagstaff et al. [6] identified six such measures. In 1997 Mackenbach and Kunst [4] listed 12 measures, and in 2005 Harper and Lynch [35] compiled more than 15 of them. The main dichotomy in the expression of the magnitude of inequalities relates to whether the measure is *absolute* or *relative.* An example of an absolute measure of inequality is the difference between the extreme wealth quintiles—for example, measles immunization coverage is 10 percentage points higher in the top wealth quintile than in the bottom quintile. A relative measure of inequality is based on a ratio—for example, vaccine coverage is 20%, or 1.2 times, higher in the richest quintile than in the poorest. The distinction between percentage points and percentages is essential. If vaccine coverage in the richest and poorest groups is 70% and 50%, respectively, the absolute difference in coverage will be equal to 20 percentage points, while the relative ratio will be 1.4 (i.e., 70%/50%), or 40% (i.e., [1.4−1]×100%).

Despite their simplicity, these measures, which take into account only the top (Q5) and bottom (Q1) quintiles of the population under study, have important limitations. First, these measures are sensitive to changes in the number of individuals in each stratification category. For example, the rich/poor ratio for coverage with skilled birth attendants based on the 2008 Nigeria DHS survey [36] is 10.4 if we use deciles of wealth and 8.8 if we use quintiles. Another limitation is that in some cases the lowest and highest wealth groups will not have the lowest and highest coverage levels, particularly when overall coverage is high. This is the case for measles vaccine coverage in Bolivia (coverage of 75% in Q1 and 67% in Q5) and Tajikistan (coverage of 88% in Q4 and 84% in Q5) [22].

More importantly, the intermediate population groups (e.g., Q2 to Q4) will not be captured in these simple measures of inequality [4],[6]. More sophisticated indicators can overcome this limitation by using information on the whole population. Harper and Lynch [35],[37] recommended the use of the absolute concentration index or the slope index of inequality (SII) as indicators of absolute inequality, and use of the relative concentration index (CIX) or the relative index of inequality (RII) as indicators of relative inequality. In this article we will focus on the SII and the CIX, which are among the most used measures of inequality in the epidemiologic and economic literature, along with ratios and differences.

The CIX is related to the Gini coefficient [6], which is widely used to measure how much income is concentrated in the hands of the richest in a given country. The Gini coefficient can be expressed in the form of a curve that shows the sample ranked by income on the *x-*axis, and the cumulative distribution of income on the *y-*axis. If everyone in the population has the same income, the curve lies exactly over the diagonal and the Gini index is equal to zero. The area between the diagonal and the observed curve is used to measure the degree of income concentration. The CIX uses an analogous approach by ranking individuals according to socioeconomic position on the *x-*axis and plotting, for example, cumulative intervention coverage on the *y-*axis. Thus, if every wealth quintile had 20% of all the vaccines distributed in a population, for example, the line would be exactly on the diagonal, and there would be no inequality [6],[35].

**Figure 2:**
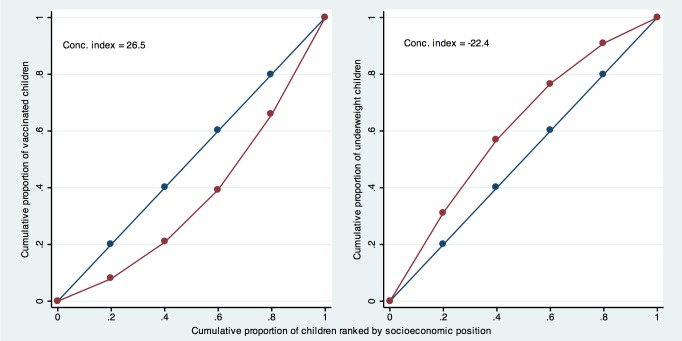
Concentration curve for measles vaccination and underweight using data from the Nigeria 2008 DHS. Conc. index, concentration index.

Typically, however, health interventions are more concentrated towards the richer groups, and the CIX assumes a positive value, as the curve is below the diagonal. Figure 2 (left) shows the example of measles vaccination in Nigeria [36], where coverage levels in the five quintiles were 17%, 28%, 41%, 58%, and 75%, respectively, and the CIX is equal to 26.5. By contrast, in the case of ill health, where poorer groups are affected more than richer groups, the CIX is negative. So in Nigeria, where underweight prevalence for the wealth quintiles Q1 to Q5 was 36%, 29%, 22%, 16%, and 10%, respectively, the CIX is −22.4 (Figure 2, right).

The main downside of the CIX is the lack of direct interpretability of its values. Clearly, a value of 20 means more inequality than a value of eight, but these numbers lack a clear meaning, unlike Q5/Q1 ratios, which are easily interpretable.

Alternative formulations for CIX can be used to reflect absolute inequalities [35], but these are used less often than the formulation we have adopted here, which reflects relative inequalities. For measuring absolute inequalities, the SII is being increasingly used [35]. This index is typically derived through linear regression of the health outcome on the midpoints of the ranks obtained by ordering the sample by the explanatory variable when using grouped data. The ranks are scaled so that the values range from zero to one. When using ranks based on quintiles, each group includes approximately 20% of the sample, and the midpoints of the ranks are close to 0.1, 0.3, 0.5, 0.7, and 0.9 for the five quintiles, respectively. The SII is the slope of the resulting regression line, and represents the absolute difference in the fitted value of the health indicator between the highest (score of 1) and the lowest (score of 0) values of the socioeconomic indicator rank. Using the same data used to calculate CIX in Figure 2 (measles vaccination in the 2008 Nigeria DHS survey) [36], we get a regression line that crosses the *y-*axis (where socioeconomic position equals zero) at 7.6% coverage, and crosses the right side of the chart (where socioeconomic position equals one) at 80%. The SII equals 72.4, which is the difference between these two coverage levels, and indicates that vaccine coverage at the top of the wealth scale is 72.4 percentage points higher than at the bottom.

There are two potential problems with a linear regression approach like this when used with an indicator, such as intervention coverage, that has a minimum of 0% and a maximum of 100%. The first is that it assumes a linear relationship between outcome and predictor, which is not always the case, particularly when a “top inequality" or “bottom inequality" pattern is present (see Box 3). Second, for rare or common outcomes, the model can fit values outside the 0%–100% interval. It can, for example, indicate negative coverage values among the poorest, which is clearly impossible. Using logistic regression instead of a linear model frequently solves both these problems. This approach allows the calculation of the difference between the estimated coverage at the top and bottom of the socioeconomic position scale and is best done using individual data rather than grouped data. In the example of measles vaccination in Nigeria, the logistic regression approach yields an SII equal to 66.8—smaller than but not greatly different from the SII obtained with the linear regression approach.

**Box 3. Patterns of Inequality**

**Figure 3:**
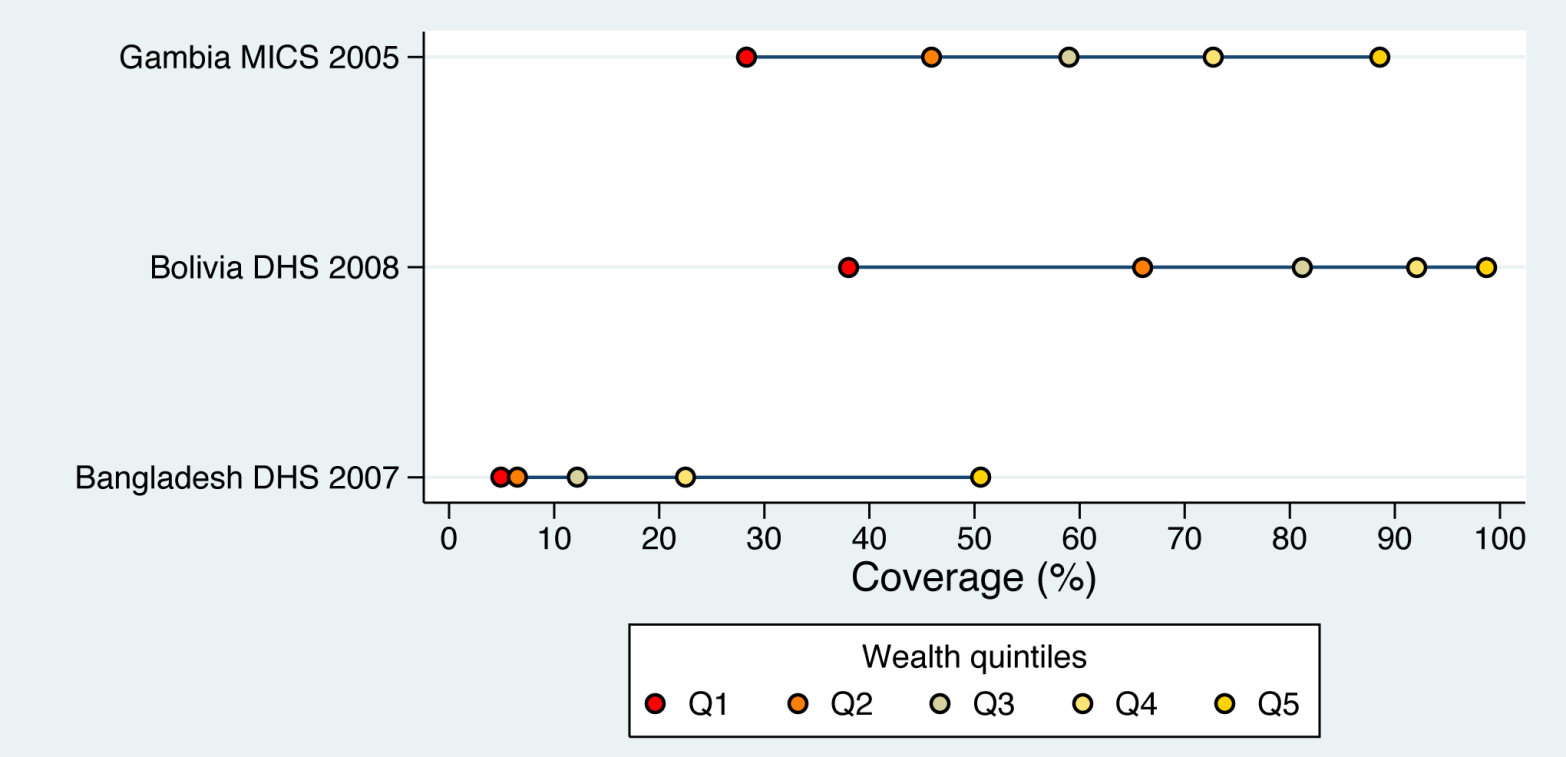
Linear, bottom, and top patterns of inequality for skilled birth attendance in Gambia, Bolivia, and Bangladesh, respectively. See Box 3 for further discussion of patterns of inequality.

- Inspecting the distance between groups in an inequality graph (such as the five-dot plots in Figure 3) can help in the design of more efficient approaches for improving coverage and reducing inequality.
- Three types of patterns of inequality have been described as “linear," “bottom" and “top" inequality patterns by Victora et al. [33] and as “marginal exclusion," “queuing," and “mass deprivation" by *The World Health Report 2005* [47]. In Figure 3, Gambia, Bolivia and Bangladesh are, respectively, typical examples of these three situations.
- Under usual conditions, low-coverage countries tend to show a top inequality pattern, with the richest quintile way ahead of the rest. As coverage increases they move to the linear pattern, where the distance between groups is similar. When higher levels of coverage are attained, a bottom inequality pattern usually appears, with the poorest lagging behind [33],[47].
- Where there is a linear pattern of inequality (Gambia, Figure 3), increased coverage in all groups is still needed, but targeting the poor should also be considered to avoid a bottom inequality pattern evolving.
- Where there is a bottom inequality pattern (Bolivia, Figure 3), targeting the poor is recommended because most of the population has already achieved reasonable levels of coverage.
- Where there is a top inequality pattern (Bangladesh, Figure 3), it is important to disseminate interventions widely, because coverage is low even in the wealthiest group.

A measure that is closely related to the SII is the relative index of inequality, or RII. The curve-fitting procedure used to calculate the RII is the same as for the SII, but instead of calculating the difference between the fitted values for one and zero, the RII is the ratio between the two. In the Nigeria measles vaccination example, the RII equals 10.6 (80% divided by 7.6%) when the linear regression approach is used. The estimate reduces to 6.4 if logistic regression is used. Given the potential problems associated with linear regression, we strongly advise that logistic regression should always be used in the calculation of SII or RII for coverage indicators.

There is near consensus in the recent literature that no single measure of inequality reveals the full picture, and that authors should report both absolute and relative measures [35],[38]. Relative measures—for example, coverage among the rich is twice as large as among the poor—give an idea of the degree of unfairness. Absolute measures—coverage among the rich is 60 percentage points higher than among the poor—give an idea of the actual effort that will be needed to close the gap. Value judgments, therefore, are implicit in the choice of measures [39], an issue that will be discussed in more detail in the next section. As an aside, there have also been discussions recently on the need to assess the pattern, or type, of inequalities, along with their magnitude, to fully understand the implications of these inequalities for health policy (Box 3).

## Trends in Inequalities

The debate on absolute versus relative measures of inequality alluded to above is particularly controversial when it relates to the issue of whether inequalities are increasing or declining over time [30],[38]–[41]. In some cases, results have been deemed inconsistent because an absolute measure indicated increased inequalities and a relative measure indicated decreased inequalities or vice versa. We show here that absolute and relative measures of inequality are not inconsistent when assessing trends, but are actually complementary.

**Figure 4:**
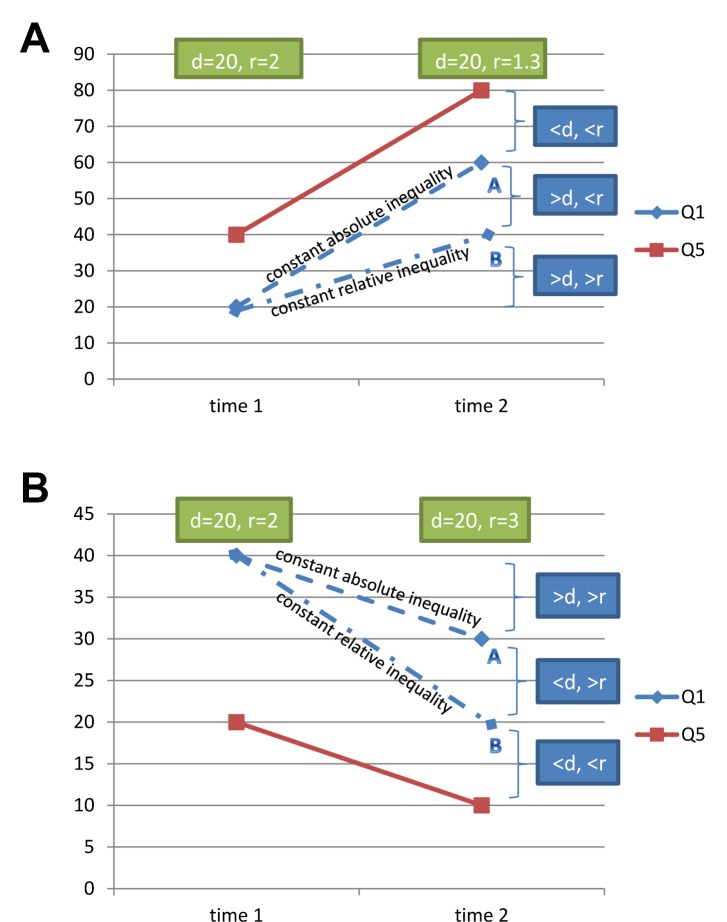
Different situations in relation to the time trend of the health indicator studied, and how changes are related to increased or decreased measures of inequality. (A) Situation 1—increasing rates of a health indicator, typical of a preventive intervention, such as immunization, or a desirable behavior such as exclusive breastfeeding. (B) Situation 2—declining rates of a health indicator, typical of an ill-health indicator, such as undernutrition or mortality, or a risk factor, such as high parity. “d” indicates the difference in coverage between the top and bottom quintiles; “r” indicates the ratio of the coverage in the top and bottom quintiles.

In Figure 4 we present two situations in which the time trend of a hypothetical health indicator is analyzed. In situation 1, the outcome indicator increases over time (e.g., the coverage of a preventive health intervention). In situation 2, the outcome decreases (e.g., mortality rate or nutritional deficit).

Let us assume that in situation 1 the richest quintile starts at 40% coverage, and the poorest at 20%. The baseline difference equals 20 percentage points, and the ratio equals two. Let us also assume that coverage among the richest increases to 80% at end point. We can then explore two alternatives for coverage among the poorest: coverage “A," where absolute inequality remains unchanged (the difference is the same as at baseline), and coverage “B," where relative inequality remains constant over time (same ratio). The worst scenario in terms of inequality is an end point for the bottom quintile where coverage is below B, because in this region of the chart, inequality will have increased both in terms of the difference and the ratio. The ideal scenario is an end point where coverage is above A. Here, both the difference and the ratio will have decreased. Finally, there are intermediate situations, where coverage is in between A and B. Here, results are apparently inconsistent. Compared to baseline, the difference between the extreme quintiles (absolute inequality) will have increased and the ratio (relative inequality) will have decreased.

In situation 2 where the outcome is declining, we have similar results for the worst- and best-case scenarios (coverage above A and below B, respectively) as in situation 1, with both the difference and ratio increasing or decreasing. The intermediate scenario, on the other hand, is different: the difference will have decreased but the ratio will have increased.

**Figure 5:**
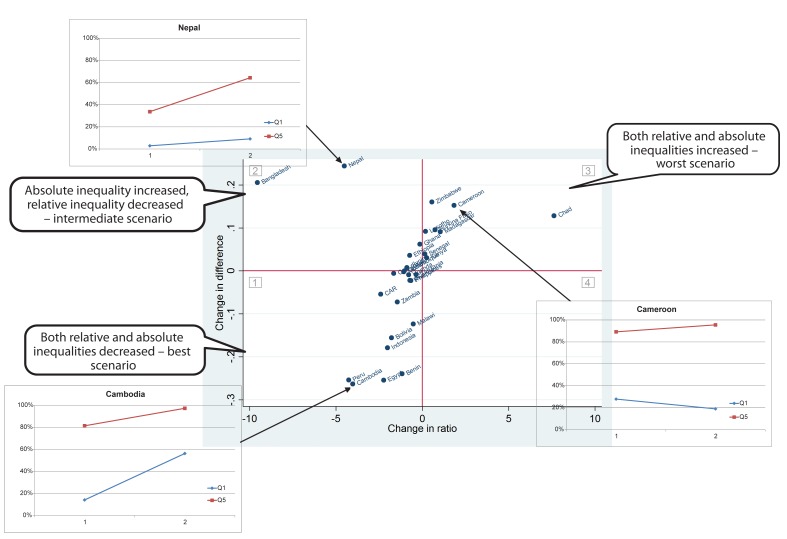
Real example of a set of countries where skilled birth attendant coverage increased over time for the richest 20% of the population. This example corresponds to situation 1 in Figure 4. CAR, Central African Republic.

In other words, the apparent conflict between changes in absolute and relative inequalities reflects scenarios where inequalities have been reduced, but not so much that both absolute and relative measures have decreased. In Figure 5 we present a real data example for 29 countries included in the Countdown to 2015 initiative where skilled birth attendant coverage increased over time for the top quintile. Quadrant 1 (lower left) represents the best scenario, where both absolute and relative inequalities decreased, with the inlaid graph showing what happened in Cambodia, from 2000 to 2010. Quadrant 2 (top left) represents the intermediate scenario, where relative inequality decreased, but absolute inequality increased, and the inlay shows the trends for Nepal, from 1996 to 2006. Finally, quadrant 3 (top right) includes countries in the worst of the situations in terms of inequality—both absolute and relative inequality increased, with Cameroon (1998–2006) as an example.

Sample variability has been often overlooked in inequality analysis, irrespective of the measure used, which is problematic when looking at trends. The convenient regression approach to the estimation of the CIX presented by O'Donnell et al. [42] is very interesting, since it allows the computation of both the point and interval estimates. A similar approach can be used to obtain confidence intervals for the SII. O'Donnell's guide and the tools provided by the World Bank [43] are invaluable additional material for those interested in analyzing and interpreting health inequalities.

## Conclusions

In this article, we have provided practical guidance on assessing inequalities in coverage of health and nutrition interventions, with emphasis on survey data from low- and middle-income countries. From our own experience, we make several recommendations about how best to assess inequalities in health intervention coverage. First, we conclude that there is no single best measure of inequality, and recommend that at least one absolute and one relative measure should be presented when describing inequalities at a given point in time, as well as when reporting trends over time. Second, when comparing time points or countries, we emphasize how important it is to calculate measures that take the whole population into account, and advocate the use of the CIX and the SII. In addition, we strongly advise the use of logit-based SII for the measurement of absolute inequalities. Because the presentation of these indices is particularly appropriate for academic audiences, we also recommend calculation of the differences and ratios among extreme quintiles, because these are easy to convey to general audiences. Third, when assessing change in inequalities, we argue that it is essential not only to evaluate both absolute and relative changes, but also to report how they evolve jointly. Finally, in situations where conflicting results are provided by absolute and relative measures, we stress that it is essential that researchers spell out the different interpretations of these measures to public health experts, because these interpretations are affected by value judgments and are likely to affect the approaches taken to reduce inequalities in the coverage of health interventions.

**Key Points**

- Recent international calls for increased accountability in measuring progress towards the Millennium Development Goals demand analyses of health indicators stratified by socioeconomic position and other equityrelated variables.
- Socioeconomic position can be ascertained using many different indicators, but the use of a wealth classification based on assets is the best option for national surveys, being feasible and reliable.
- Intervention coverage can be assessed by individual indicators, but because combined measures are important for the study of time trends and for cross-country comparisons, we advocate the use of the composite coverage index and the co-coverage indicator.
- Inequality measures that take the whole socioeconomic distribution into account are essential, and at least one absolute (the slope index of inequality) and one relative (the relative concentration index) measure should always be presented.
- When analyzing inequality trends, absolute and relative inequality must be studied jointly because there is a clear interacting pattern of reduction or increase in inequality that sometimes produces apparently contradictory changes in absolute and relative inequality.

## Abbreviations

CCI: composite coverage index
CIX: concentration index
DHS: Demographic and Health Survey/s
MICS: Multiple Indicator Cluster Survey/s
MNCH: maternal, newborn, and child health
RII: relative index of inequality
SII: slope index of inequality
